# Editorial: Family-centered care in pediatric and neonatal critical care settings

**DOI:** 10.3389/fped.2024.1402948

**Published:** 2024-03-28

**Authors:** Jos M. Latour, Janet E. Rennick, Agnes van den Hoogen

**Affiliations:** ^1^ Faculty of Health, University of Plymouth, Plymouth, United Kingdom; ^2^ Department of Nursing, Zhongshan Hospital, Fudan University, Shanghai, China; ^3^ The Curtin School of Nursing, Curtin University, Perth, WA, Australia; ^4^ Department of Nursing, The Montreal Children’s Hospital, McGill University Health Centre, Montreal, QC, Canada; ^5^ Ingram School of Nursing, Faculty of Medicine and Health Sciences, McGill University, Montreal, QC, Canada; ^6^ Department of Pediatrics, Faculty of Medicine and Health Sciences, McGill University, Montreal, QC, Canada; ^7^ Department Woman and Baby, Wilhelmina Children’s Hospital, University Medical Centre Utrecht, Utrecht, Netherlands; ^8^ Clinical Health Science, Utrecht University, Utrecht, Netherlands

**Keywords:** family-centered care, infant, child, parents, critical care, outcome measures

**Editorial on the Research Topic** Family-centered care in pediatric and neonatal critical care settings

## 1 Introduction

The impact of childhood critical illness on the child and family is profound and potentially long-lasting (1–5). It has become increasingly clear in recent decades that the child's illness can impact the psychological health of family members which, in turn, can impact the health outcomes of the child (6–9). The interdependent nature of family members' health outcomes on the child's long-term recovery was conceptualized in the Post Intensive Care Syndrome in pediatrics (PICS-p) framework developed by Manning et al. (5). Evidence supporting PICS-p underscores the importance of patient and family-centered care (FCC) in neonatal intensive care units (NICUs) and pediatric intensive care units (PICUs), resulting in a shift in focus within the global critical care community towards optimizing health outcomes for all family members.

Family-centered care is an approach to the planning, delivery, and evaluation of health care that has been widely adopted. It is grounded in mutually beneficial partnerships among health care providers, patients, and families, and includes four core dimensions: dignity and respect, information sharing, participation in care and decision making, and collaboration (10). Healthcare professionals need to acknowledge and respect the needs of patients and parents, including their cultural and spiritual backgrounds. As caregiving partners in pediatric and neonatal critical care settings, parents work closely with the multidisciplinary team to improve their child's care. Doctors, nurses and allied health professionals must recognize the importance of incorporating FCC into daily practice, however evidence suggest that they do not consistently do so (11). Consequently, measures that assess family needs, experiences, satisfaction and health outcomes have received limited attention in the field.

There is growing interest around the world in establishing standardized FCC outcome measures. The EMPATHIC questionnaire was developed to assess parent satisfaction and experiences in pediatric and neonatal intensive care settings and has been translated and validated in multiple languages and continues to be adapted for use with different cultures around the world (12). Others have conducted intervention studies aimed at improving FCC practices (13–16). In related work, in 2020, Fink et al. (17) developed a PICU Core Outcome Set (COS) and PICU COS-Extended using an inclusive stakeholder approach (clinicians, researchers, family members) with participants from six continents to help ensure that outcomes considered important to stakeholders are also considered by clinicians and researchers seeking to improve child and family health outcomes. While both outcome sets include global domains of cognitive, emotional, and physical function, and overall health, the long-term impact of pediatric critical illness on family members' emotional health is highlighted in the PICU COS, while family functioning and post-traumatic stress in children and parents are also included in the PICU COS-Extended. The PICU Core Outcome Measurement Set was subsequently identified to evaluate PICU COS domains (18). As this work, continues to grow, so will our understanding of the provision and impact of FCC in critical care settings.

It was over a decade ago that the American College of Critical Care Medicine Task Force recommended including the family as an integral part of the intensive care unit (ICU) team (18). Those practice recommendations were based on evidence suggesting that FCC facilitates timely restoration of health, improves patient and family experiences, reduces staff stress, and optimizes the dying process (18). Despite this, a subsequent review of the literature to update critical care practice guidelines supporting FCC in neonatal, pediatric and adult ICUs revealed that progress in the field has remained limited (19).

## 2 Family-centered care in PICU and NICU

This Frontiers in Pediatrics special research topic of FCC in pediatric and neonatal critical care settings highlights important research findings in the field of FCC. The aim of this Research Topic is to build upon the current evidence and extend the body of knowledge related to the care of infants, children and their parents and family members. This Research Topic is specifically organized to contribute to a framework for action to implement effective FCC interventions and to provide evidence of evaluating current FCC practices in neonatal and pediatric intensive care settings.

## 3 Research progress in family-centered care

This e-book of the research topic *FCC in pediatric and neonatal critical care settings* includes a variety of studies using various methods and designs. Most articles report on interventions or explorations of Family-Centered Care in NICU and PICU settings. In total, 16 articles are included in this e-book. The research designs of these articles include both qualitative and quantitative research methodologies. Additionally, one articles is a scoping review and one articles is a commentary on one of the included research papers. The research projects in this research topic can be clustered in four main themes:

1. Clinical Practice: 4 articles, including one study protocol and a training program.
2. Implementation: 4 articles.
3. Family Experiences: 5 articles.
4. Outcome Measurers: 3 articles.

### 3.1 Clinical practice

Colleagues from the US (Lake et al.) explored the relationship between parent satisfaction and missed nursing care in the NICU. In this cross-sectional design study, including 30 NICUs, the authors used the adapted EMPATHIC-38 instrument and missed care was measured by a self-reported survey using the National Database of Nursing Quality Indicators including 30 variable items. High quality nursing care such as counselling, teaching, helping breastfeeding mothers, and preparing parents for discharge were identified as important. Missing these care activities might influence parent satisfaction. Parent satisfaction, and specifically related to care and treatment, is related to missed nursing care.

A scoping review from Ullsten et al. was performed to obtain a deeper understanding of the evidence of parent-driven pain- and stress-relieving interventions in neonatal care. Of the 93 included articles, the synthesis of the findings suggested that the efficacy of skin-to-skin contact and breastfeeding, preferably in combination provides strong evidence in relieving pain and stress in infants. There is, however, limited evidence available on parents' motivations or experiences of alleviating infant pain. The authors conclude that advantages of involving parents in pain management are not only benefiting infants and parents but also the wider healthcare system.

Regarding the FCC clinical practices in the PICU, two articles in this e-book are included and originate from Germany and Tanzania. One article is a study protocol, and a second article is a pre-post intervention study. Ferentzi et al. described a study protocol regarding the relationship of FCC with parent and infant well-being in a German pediatric cardiac intensive care unit (PCICU). The aim of the study was to explore FCC practices in two PCICUs and to compare parent satisfaction with FCC, unit adherence to FCC practices and infant outcomes. The fourth article in this theme reports an evaluation of a neonatal resuscitation training program for healthcare professionals in Zanzibar, Tanzania (Ding et al.). The implementation of the neonatal resuscitation training program increased the theoretical knowledge and resuscitation skills before and after two training sessions and over time after a 9-month period. Adequate training among healthcare professionals is important to increase infants' well-being and health outcome. How parents were involved in training is not known from this study but it is important to address and include parents in future training programs for healthcare professionals. To ensure parents are an integral part of the neonatal team, and to become confident caregivers for their infant both in the neonatal unit and after discharge, parents should be offered education, training, and support in specific skills (EFCNI standards of care: https://newborn-health-standards.org/standards/standards-english/education-training/education-programme-supporting-parents-and-families/).

### 3.2 Implementation

In the Netherlands, Oude Maatman et al. aimed to identify factors influencing implementation of FCC in a NICU. The authors found that the mind-set of healthcare professionals in seeing parents as primary caregiver influences the way FCC is practiced and how parents are supported in their involvement in the care of their infant. Subsequently Cruz et al. (20) wrote a commentary on this study and stated that it is crucial to provide healthcare teams and managers with knowledge about FCC that emphasizes the importance of interprofessional practices: “The recommendation of Oude Maatman and colleagues to initiate FCC implementation with staff recognition of parents as primary caregivers constitutes a strategy for promoting FCC in NICUs in various healthcare systems” (Oude Maatman et al.).

Changes of infant- and family-centered developmental care practices administered to extremely preterm infants during the implementation of the Neonatal Individualized Developmental Care and Assessment Program (NIDCAP) program was studied by Klein et al. Data were collected regarding clinical and caring procedures during the first 14 days of life and included NIDCAP observations, infant pain management, skin-to-skin contact, and family access and involvement in the care. The authors found statistically significant improvements in multiple important outcome measures in all hospitalized extremely preterm infants when implementing NIDCAP. Overall, implementing NIDCAP might positively influence the FCC practices which might lead to prevention of pain, increased parental involvement in care and increased skin-to-skin care.

In a PICU setting, Pereira et al. from Brazil conducted a mixed-methods program evaluation targeting perspectives and feedback from PICU families and healthcare professionals. The goals were to support: (a) families to adjust with the PICU experience with the support of a peer mentor and (b) children to receive non-medical interaction from trained volunteers. The evaluation served as a change management strategy and in addition helped to identify areas for improvement and strategies. The results of this program evaluation might help healthcare professionals to identify the benefits of their caring duties described in their job description and how their capacity influence the emotional support and guidance to families related to FCC practice.

### 3.3 Experiences

In a qualitative study, Ferreira et al. explored the experiences of parents that either helped or hindered in providing care to their infant in the NICU. Nine mothers and one father were interviewed three times over a 3-month period in small focus groups. Eight themes were identified from the qualitative data and a conceptual framework was developed around the topic of parental involvement including the themes: parent-staff interactions; supportive/trustworthy healthcare professionals; consistency in care and caring staff; family, couple, and peer support; newborn status; resources and education for parents; NICU environment; and academic and research participation. Data on parent satisfaction can guide health care professionals in developing and implementing strategies to improve parental involvement in care.

Fathers of preterm infants and their role in the NICU was debated by Baldoni et al. using data of contemporary research. Following an attachment perspective, the authors analyzed the role of the father in caring for their preterm infant within the context of the family. Involving fathers in the NICU as early as possible is essential because it provides a basis for the father/child attachment relationship. The authors suggested that being inclusive in FCC practices and supporting fathers might have positive effects on the psychological and somatic development of infants as well as the health of mothers and the wider family. In addition, having a psychologist included in the NICU team was recommended. The article provided further recommendations to promote involvement of fathers in the care of their infant.

Three qualitative research articles included in this e-book highlight the importance of supporting the family's psychological recovery during and following PICU hospitalization. One study focused on parents satisfaction with FCC in a PICU using data gathered from open-ended questions on the EMPATHIC questionnaire (Terp et al.). Using thematic analysis, the authors identified participation in decision-making about care and treatment as well as person-centered communication improved FCC. A second, descriptive qualitative study among eight PICUs in Switzerland explored sources of stress, family functioning and needs of families with a chronic critically ill child in the PICU (21). Parents were found to experience high emotional intensity and PICU-related sources of stress, family needs evolved over time, and there were multi-faceted family functioning present throughout their lives. Overall, parents' experiences were highly dependent on the abilities of PICU staff to meet their needs and support their wishes to be empowered in their parenting, and to share in the delivery of high-quality care to their children. In the third paper, an interpretive descriptive design was used to explore children's and parents' perceptions of their psychological and behavioral responses to PICU hospitalization one-year post-discharge (Rennick et al.). Families from three Canadian PICUs described ongoing efforts to adapt and reestablish a sense of normalcy one-year following discharge. Family members' psychological responses to PICU hospitalization were closely interrelated, highlighting the importance of viewing the family as an interdependent unit when examining and seeking to prevent long-term psychological sequelae following critical illness.

### 3.4 Outcome measures

The importance of outcome measures is evident in clinical practice in all healthcare settings including PICU and NICU. Within FCC intervention trials, several tools are available. One of these is the EMPATHIC (EMpowerment of PArents in THe Intensive Care) questionnaire, the outcome measure of parent satisfaction used in studies included in this e-book (22–24).

Colleagues adapted the EMPATHIC-30 questionnaire and constructed a new measure for use in the USA across 30 NICUs using the rigorous COSMIN criteria (Lake et al.). The authors revised and evaluated the reliability and validity of the new 38-item EMPATHIC-NICU-USA. Data from 282 parents revealed adequate reliability (Cronbach's Alpha of >0.70), good positive associations between the five domains of the instrument. The expanded and newly named instrument, EMPATHIC-38-NICU-USA, revealed satisfactory psychometric properties and the authors plan to use this instrument as an outcome measure in their future trials.

In another study, Tiryaki et al. in Turkey translated and validated the EMPATHIC-30 questionnaire to measure parent satisfaction in the NICU. Besides using Cronbach's Alpha, the authors performed further analyses on their sample of 238 responses using the intraclass correlation coefficient (ICC = 0.998) in the test-retest evaluation and the confirmatory factor analysis, which resulted in a moderate fit. The authors concluded that the Turkish version of the EMPHATIC-30 is an easy-to-use outcome measure of parent satisfaction in the NICU.

A third translation and validation study is included in this e-book in which the EMPATHIC-N was used to measure parents satisfaction in three NICUs in Ethiopia (Gulo et al.). The EMPATHIC-N was translated into two languages used in Ethiopia. The psychometric properties of both translated EMPATHIC-N versions were moderate based on a sample of 386 responses. The authors also observed differences in parent satisfaction outcomes across the three NICUs. This highlights the importance of using standardized outcomes measures to be able to benchmark outcomes across multiple centers, regionally, nationally, and internationally.

## 4 Towards a core outcome set for family-centered care

NICU- and PICU-based FCC interventions have demonstrated a positive impact on the health outcomes of infants, children, parents and other family members. Yet, despite a growing body of evidence regarding the implementation of FCC interventions, the reporting of varied outcomes and the use of diverse outcome measures among infants, children, and parents limits the usefulness of these studies in improving healthcare services (25). Recently, a systematic review and meta-analysis conducted by Ding et al. (26) identified several FCC interventions and outcome measures within neonatal trials. The different outcomes reported in this review demonstrate variability in the use of multidimensional models of care for FCC interventions in clinical trials. Combining and comparing the effect of different outcomes and outcome measurements in clinical trials is important to test the effectiveness and safety of FCC interventions. This systematic review and meta-analysis identified the challenges of using standardized outcome measures and instruments. For example measuring parental stress and anxiety was measured by several different outcome instruments to test FCC interventions (Tiryaki et al.).

The Core Outcomes In Neonatology project (COIN) emphasized that there was inconsistency in reported outcome measures in NICU studies (27). This drew attention to the lack of evidence to draw upon in standardizing a core outcome set and related core outcome measurement set in neonatal care. While both are important to promote higher quality FCC intervention trials in the NICU, they have not yet been developed. The COUSIN study is one of the initiatives underway to strengthen the evidence of FCC intervention trials in the NICU and ultimately support FCC practices (28). The aim of this study is to develop a core outcome set with outcome measures to evaluate FCC practices in NICUs. The objectives in developing the core outcome set are aligned with the methodology described in the COMET Handbook (29).

## 5 Summary

This e-book, with a collection of 16 articles, covers the latest evidence regarding the science and practice of FCC in pediatric and neonatal critical care settings. The diversity of research conducted to-date indicates a high level of engagement of clinical and academic staff, and family partners working to improve PICU- and NICU-FCC practices. Current evidence also indicates a need for standardization of FCC interventions and ways to evaluate these interventions such that the results of any study become meaningful to all stakeholders. Intrinsically, core outcome sets with standardized outcome instruments can increase research engagement of all stakeholders, including parents and former patients, and strengthen the evidence base of FCC practices in PICUs and NICUs. These core outcomes can also support the implementation of best practices as identified by the current research articles in this e-book. Further research and quality improvement projects are needed to support FCC practices across the world to improve the physical, cognitive, emotional and social health of infants, children, young people and their families.

It is time to reunite with clinicians and academics in pediatric and neonatal intensive care to drive the health and research agenda forward together with our patients and families.
